# Recent Advancements in Pathogenic Mechanisms, Applications and Strategies for Entomopathogenic Fungi in Mosquito Biocontrol

**DOI:** 10.3390/jof9070746

**Published:** 2023-07-13

**Authors:** Yujie Qin, Xiaoyu Liu, Guoxiong Peng, Yuxian Xia, Yueqing Cao

**Affiliations:** 1 School of Life Sciences, Chongqing University, Chongqing 401331, China; 2 Chongqing Engineering Research Center for Fungal Insecticides, Chongqing 401331, China; 3 Key Laboratory of Gene Function and Regulation Technologies, Chongqing Municipal Education Commission, Chongqing 401331, China

**Keywords:** mosquito, entomopathogenic fungi, biocontrol, pathogenicity

## Abstract

Fungal diseases are widespread among insects and play a crucial role in naturally regulating insect populations. Mosquitoes, known as vectors for numerous infectious diseases, pose a significant threat to human health. Entomopathogenic fungi (EPF) have emerged as highly promising alternative agents to chemical mosquitocides for controlling mosquitoes at all stages of their life cycle due to their unique infection pathway through direct contact with the insect’s cuticle. In recent years, significant advancements have been made in understanding the infection pathways and pathogenic mechanisms of EPF against mosquitoes. Various strategies involving the use of EPF alone or combinations with other approaches have been employed to target mosquitoes at various developmental stages. Moreover, the application of genetic technologies in fungi has opened up new avenues for enhancing the mosquitocidal efficacy of EPF. This review presents a comprehensive summary of recent advancements in our understanding the pathogenic mechanisms of EPF, their applications in mosquito management, and the combination of EPF with other approaches and employment of transgenic technologies. The biosafety concerns associated with their use and the corresponding approaches are also discussed. The recent progress suggests that EPF have the potential to serve as a future biorational tool for controlling mosquito vectors.

## 1. Introduction

Mosquitoes pose a significant global threat as they serve as vectors for transmitting various infectious diseases, such as malaria, yellow fever, dengue, chikungunya, West Nile fever, and Zika fever [1,2]. According to the data provided by the World Health Organization, malaria presents a substantial risk to approximately half of the global population, with an estimated annual infection rate of 200–300 million individuals and an alarming mortality rate of almost one million per year [3].

So far, chemical insecticides have served as the primary method for controlling and eliminating mosquitoes [4,5]. Nevertheless, the excessive use of synthetic insecticides has resulted in mosquito resistance and poses significant risks to the environment and non-target organisms, including humans [6]. As a result, there has been an increasing focus on exploring chemical-free biocontrol approaches to address these challenges. These approaches include the use of bacteria, viruses, and fungi as more comprehensive vector control interventions [7].

Bacteria and viruses can play a role in the digestive tract when ingested by insects. However, their effectiveness in controlling adult mosquitoes with piercing–sucking mouthparts is limited [7,8]. Entomopathogenic fungi (EPF), on the other hand, can infect mosquitoes through direct contact with the mosquito’s cuticle, without the need for ingestion, making them highly attractive as control agents. These fungi are ecologically safe and have the capacity to target mosquitoes at all stages of their life cycle, including adults, eggs, larvae, and pupae [9,10,11,12,13,14]. Additionally, EPF have long-lasting effects on the developmental parameters of mosquitoes, such as reduced fecundity in subsequent generations [15].

In recent years, EPF have been increasingly employed for mosquito control in the field [16,17]. Additionally, various other approaches, including insect attractants, chemical insecticides, microbial metabolites, predators, and other microbial pesticides, have been integrated with EPF applications, showing a synergistic effect in mosquito control [18,19,20,21,22,23,24]. The mechanisms underlying mosquito control by fungi have been extensively investigated, and genetic engineering techniques have provided novel insights and strategies for effective mosquito control [4]. This article presents a comprehensive review of recent advancements in understanding pathogenic mechanisms, the application of EPF at different life stages of mosquitoes, the integration with other approaches, and the use of the transgenic technologies in mosquito control.

## 2. Pathogenic Mechanisms of EPF and Immune Response of Host Mosquito

**Figure 1:**
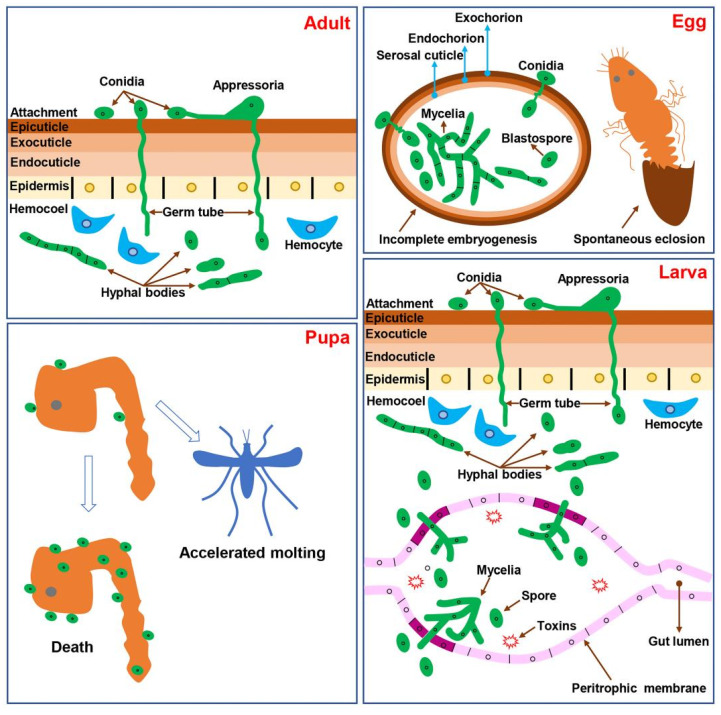
Two invasion pathways of EPF in mosquitoes: cuticle and digestive tract routes. EPF infect mosquito adults, eggs, and pupae through contact with the cuticle. Larvae can be infected through both the cuticle and digestive tract routes. Fungal conidia attach to the cuticle and germinate to form germ tubes, which either penetrate the cuticle directly or develop appressoria at the end of the germ tubes to penetrate the cuticle. Once the cuticle is breached, hyphal bodies rapidly developed in the hemolymph of adult. In eggs, the growth of hyphal bodies inside can cause incomplete embryogenesis or spontaneous premature eclosion. Toxic metabolites can disrupt the balance of microorganisms in the midgut and accelerate mosquito mortality. In pupae, cuticle infection can lead to death or accelerated molting. In larvae, similar to adults, conidia can infect through the cuticle and enter the hemolymph. Additionally, conidia can be ingested and grow within the digestive tract. The fungus can penetrate the peritrophic membrane of the midgut to enter the hemolymph.

There are two invasion pathways for EPF to invade a mosquito: invasion through cuticle and invasion through the digestive tract by ingestion. EPF primarily target eggs, pupae, and adults through contact with the cuticle, while for mosquito larvae control, both of the invasion pathways have been reported. The two ways the fungus can invade the mosquito adult, egg, pupa, and larva are summarized in Figure 1.

### 2.1. Invasion through Mosquito Cuticle

The typical infection processes of EPF occurs through contact with mosquito cuticle. Fungal conidia attach to the mosquito’s surface, germinate to form germ tubes, and develop appressoria at the end of germ tubes. They then penetrate the host’s cuticle through a combination of mechanical force from turgor pressure and enzymes produced by the appressoria. This penetration allows the fungi to destroy the insect immune system and proliferate inside the insect [25,26,27]. Infection through the cuticle is undoubtedly the main pathway for EPF to target eggs, pupae, and adult mosquitoes with piercing–sucking mouthparts. The fungal infection can lead to the death of the mosquito or disrupt its further development, such as eclosion, pupation, oviposition and reduced life span [13,25,28,29,30,31]. EPF infection on adult mosquito has been extensively described, showing a similar phenomenon as other insects [27]. Similar as in adults, topical infection of larvae can destroy the host hemocytes after penetrating the cuticle [32]. However, the ingestion pathway has been reported to serve as the main infection route for larvae control [5]. When eggs are treated, conidia can adhere to the chorion, germinate, and form germ tubes and appressoria. They then penetrate the chorion of egg, resulting in the growth of mycelia and new conidia on the egg surfaces. This growth disrupts the eggshells and halts the development of the embryo within the eggs [29]. In some cases, fully developed larvae in the eggs may be stimulated to eclose prematurely when exposed to conidia or when invaded by hyphae. This causes a higher rate of spontaneous eclosion as a mechanism for escaping from the fungal infection. However, it is worth noting that spontaneous eclosion does not occur when fungal growth takes place on the surface of the eggs, indicating a cessation in embryo development when this phenomenon occurs [13,29]. When applied to pupae, blastospores of *Metarhizium anisopliae* can secrete mucilage to facilitate adhesion of the fungus to the insect’s integument, which accelerates infection and reduces the time to kill the mosquito [33]. After the pupae succumb to infection by *M. anisopliae*, the mosquitoes accelerate the molting process to the adult stage, trying to escape the infection [33].

The molecular mortality mechanisms of pathogenic fungi have been extensively explored in recent years, leading to some new discoveries. In the surface invasion pathway, the mechanical pressure exerted by fungal infection structure appressorium is essential for the penetrating mosquito cuticle. Understanding these mechanisms behind appressorium turgor generation may provide strategies for improving fungal biopesticides. The histone methyltransferase ASH1 and peroxidase Mrpex16 pathways (ASH1-PEX16) play an essential role in regulating the biogenesis of peroxisomes, which promote lipid hydrolysis to produce large amounts of glycerol for turgor generation in appressoria [25]. The histone lysine methyltransferase KMT2-Cre-Hyd4 pathway has also been found to participate in the regulation of lipid and carbohydrate metabolism and transportation to facilitate fungal infection [34].

Mosquito-associated microbiota also play a vital role in the development, survival, and immunity of mosquitoes [7,35]. They can suppress the mosquito’s innate immune system to favor *Plasmodium* infection and possibly malaria transmission [36]. Topical fungal infection can break down the immune system of mosquitoes, leading to an increase in microbiota abundance, a decrease in bacterial diversity in the midgut, and accelerated mosquito mortality [37]. Recently, a new fungal pathogenic mechanism was discovered for mosquito larvae. During the infection process, *Beauveria bassiana* exports a microRNA-like RNA (bba-milR1) that hijacks the host’s RNA-interference machinery in mosquito cells to suppress the host immune defense, including the expression of antimicrobial peptide genes and melanization [38]. Additionally, insect hosts also transfer their miRNAs to the fungal pathogen in order to suppress fungal infection [39].

### 2.2. Invasion through Digestive Tract

In addition to the typical pathway of cuticle penetration, an alternative pathway involving ingestion through the digestive system has been observed in mosquito larvae. In the case of *M. anisopliae*, the conidia fail to adhere to the cuticle of *Aedes aegypti* larvae, thereby preventing normal invasion and pathogenesis [40]. A similar phenomenon of failure to attach to the larvae’s body surface has also been observed in *Aspergillus clavatus* [41]. The Pr1, a cuticle-degrading subtilisin protease essential for cuticle penetration, is not induced when conidia are applied to *Ae. aegypti* larvae, suggesting that fungi have an alternative means of invading mosquito larvae [40,42]. When *B. bassiana* conidia are suspended in water, they are primarily localized in the gut, mouthparts, and perispiracular lobes of *Ae. aegypti* larvae [43]. Fungal conidia can obstruct the midgut of *Ae. aegypti* larvae, leading to a decrease in the total hemocyte concentration and the inability to stimulate the phenoloxidase activity, which is a proxy for the general activation of the innate immune system of the insects. This downregulation of the immune response also downregulates the expression of antimicrobial peptides, enabling the fungus to impair the larvae and facilitate infection [44]. Furthermore, both *B. bassiana* and *M. anisopliae* can invade the hemocoel from the midgut of *Ae. aegypti* larvae [44,45].

In the ingestion pathway, toxic molecules play a crucial role in the pathogenesis of host mosquitoes. Certain secondary metabolites produced by EPF have been discovered to be toxic to mosquitoes. Metabolites derived from ethyl acetate in *M. anisopliae* and *B. bassiana* have been found to be effective against mosquito larvae while exhibiting lower toxicity effects on non-target organisms [46,47,48]. These metabolites have shown high toxicity against larvae, pupae, and adults of *Ae. aegypti*, *Anopheles stephensi* and *Culex quinquefasciatus* mosquitoes [48,49]. In the case of *A. clavatus*, ingested conidia accumulate in the digestive tract of the larvae, causing tissue disorders and leading to the death of the mosquito through the release of fungal metabolites [41]. Similarly, ingested *Pythium guiyangense* conidia can kill *Aedes albopictus* and *Culex pipiens pallens* by secreting proteases and kazal-type protease inhibitors in the midguts of mosquitoes. These inhibitors hinder larval food digestion [10]. These findings demonstrate that in addition to entomopathogenic fungal conidia, their metabolites and secretions hold potential as effective, cost-efficient, biodegradable, target-specific alternatives to chemical insecticides in mosquito control programs.

### 2.3. Immune Response of Mosquito

When invasion occurs, the mosquito defends against the pathogens by both physical and physiological barriers. After pathogens break through the host’s physical barriers, such as cuticular and epithelial barriers (epidermal, intestinal, and tracheal networks), and reach the hemocoel, the innate immune system of insects is triggered. Unlike higher organisms, insects lack an adaptive immune system. However, their well-developed innate immune system offers a general and rapid response to pathogen infection [50]. The insect innate immune system relies on cellular responses (phagocytosis, nodulation, and encapsulation) as well as humoral responses (antimicrobial peptides, melanization and reactive oxygen species) [43,51]. Humoral responses of mosquitoes against fungi are largely induced through Toll, JAK-STAT, and IMD pathways [52]. Previous extensive reviews have covered the mosquito’s innate immune pathways and response to entomopathogenic fungi [50,51]. The roles of components in these pathways are conserved in insects and have gradually been clarified to involve immunity against mosquitoes. A recent study demonstrated that an OTU7B protein can block the *Ae. aegypti* immune response to *B*. *bassiana* infection by removing the polyubiquitin chains of the Toll adaptor TRAF4 [53]. Furthermore, studies have indicated that when infected by EPF, mosquito can generate miRNAs that migrate into fungal cells and silence genes associated with virulence, thereby reducing fungal pathogenicity [54].

## 3. Effectiveness of EPF in Mosquito Control

### 3.1. Effectiveness of EPF on Different Development Stage of Mosquito

*Metarhizium* and *Beauveria* are two main generalist entomopathogenic fungi that have been widely used in pest control in various insect species, including agricultural pests and mosquitoes [26]. The recent advancements in the application of EPF in mosquito control has been extensively reviewed by Cafarchia et al., focusing on the field application of formulations of *B. bassiana* and *M. anisopliae* and providing detailed information on these two main fungal strains in mosquito control [8]. Additionally, Shen et al. also provide a summary of the application of EPF on mosquito larvae and adults [5]. In recent years, EPF have been found to affect mosquito development and can also effectively control mosquito at the egg and pupal stages. In this context, we have compiled a summary of recent applications and effectiveness of various entomopathogenic fungal strains in biocontrol of mosquitoes at different life stages, as presented in Table 1.

### 3.2. Factors That Influence Spore Quality

The efficacy of fungal strains in biocontrol can vary among different mosquito species, and spore quality plays crucial for the biocontrol effectiveness. The choice of culture media also has an impact on the virulence of fungal conidia against *Ae. aegypti* larvae. Conidia produced on rice grains have demonstrated higher virulence compared to those cultivated on artificial media such as RYA and SDA [12]. Furthermore, the *Metarhizium brunneum* blastospores exhibit higher virulence toward *Ae. aegypti* larvae than conidia, due to multiple routes of entry (cuticle and gut) in water [42]. A recent report indicates that *M. anisopliae* blastospores exhibit higher virulence against *Ae. aegypti* adults, larvae and pupae [11,33,42,45]. Supplementation of Riboflavin and NaNO_3_ in the culture medium has been shown to enhance protease and conidial production, leading to improved larvicidal activity against *Ae. aegypti* [22]. Additionally, mineral oil has been shown to enhance the efficacy of fungal propagules in the aquatic environment, demonstrating its potential as an adjuvant in entomopathogenic fungi [43]. These studies highlight the importance of selecting the appropriate form of inoculum and cultural condition for efficacious control of disease vectors.

## 4. Combination of EPF with Other Strategies in Mosquito Control

A singular method or intervention is often insufficient to effectively control vector-borne diseases, and therefore a holistic and integrated approach is necessary. Integrated vector management (IVM) is a comprehensive approach for mosquito control that combines multiple vector control methods and approaches in a coordinated manner. This includes source reduction, as well as larvicidal and adulticidal applications to control mosquitoes at different life stages [24]. IVM offers several advantages by integrating multiple control strategies, resulting in effective prevention and measures. Consequently, it is well-suited for large-scale mosquito- and insect-control efforts. EPF have increasingly been employed for effective field control of mosquitoes, targeting eggs, larvae, pupae, and adults [8,33]. The utilization of EPF in conjunction with other mosquito control strategies has demonstrated a synergistic effect and have the potential to further increase the efficacy of IVM program for mosquito control.

### 4.1. Combined with Chemical Insecticides

Chemical insecticides often have specific targets, making mosquitoes prone to developing resistance [73,74]. Mosquito resistance is typically associated with the induction of detoxification enzymes,, including cytochrome P450 monooxygenases, acetylcholinesterase (AChE), glutathione S-transferase (GST), esterase (EST), acid phosphatases (ACP), and alkaline phosphatases (ALP) [74,75,76]. Fungal insecticides, on the other hand, can diminish the immune defenses and reduce the activity of detoxification enzymes in mosquitoes. Studies have shown that *M. anisopliae* and *B. bassiana* can suppress the enzymatic activities of ACP in chlorpyrifos-resistant *Cx. quinquefasciatus* [75]. *Metarhizium anisopliae* is compatible with diflubenzuron at lower concentrations and combined applications have shown to enhance *Cx. pipiens* management [77]. Furthermore, the combination of *M. anisopliae* with the insecticide Imidacloprid (IMI) increases virulence against *Ae*. *aegypti* when ultra-low concentrations of IMI are used [78]. Hence, fungal mosquitocides effectively combat mosquito populations that have developed resistance to certain chemicals or drastically reduce the consumption of chemical pesticides.

### 4.2. Combined with Microbial Metabolites or Microbial Organisms

Many microbial metabolites, such as avermectins, a type of neurotoxic insecticide, and Asperaculane B, which can inhibit the acetylcholinesterase enzyme, have been extensively employed as effective biocontrol insecticides [19,21,79]. Combining insect pathogenic fungi with microbial metabolites represents a promising approach to mosquito control. The co-application of *M. robertsii* and avermectins lead to a synergistic effect on *Ae. aegypti* larvae mortality [21,80]. Avermectins can reduce the relative abundance of antagonist in mosquito gut, favoring the fungus [80]. *Metarhizium robertsii* significantly reduces the activity of detoxification enzymes, such as esterases, proteases, and phenoloxidase in mosquitoes, disrupting the immune and detoxifying systems and promoting fungal infection [21,80]. *Bacillus thuringiensis* (Bt) has been extensively studied and commercially applied in pest control due to the high pesticidal activity of Bti endotoxins [20,81]. The combined application of the mosquito larvae pathogen *Leptolegnia chapmanii* with Bt produce a synergistic larvicidal effect on *Ae. aegypti* [20]. Additionally, many EPF have been found to produce metabolites toxic to mosquitoes. Twelve metabolites from *Penicillium toxicarium* extracts exhibited high toxicity to mosquito larvae and adults [82]. *P*-orlandin, a nontoxic metabolite from *A. niger*, can target mosquito FREP1, which is a critical protein for parasite infection in *Anopheles gambiae* and could block malaria transmission [83]. Several fungal cell culture filtrates have displayed mortality against mosquito [84]. Recent research on fungal metabolites in mosquito control is summarized in Supplementary Table S1. Consequently, the combination of fungal pathogens with microbial metabolites or other microbes producing toxic metabolites demonstrates a synergistic effect and reduces the reliance on chemicals in mosquito management.

### 4.3. Combined with Mosquito Attractants

The utilization of volatile compounds and semiochemicals that attract mosquitoes has been incorporated into complementary vector control strategies to enhance the effectiveness of fungal mosquitocides [22]. Numerous volatile organic compounds (VOCs) with mosquito attractant properties have been identified and analyzed, including natural hosts, chemical compounds, synthetic blends of compounds, and plant odors [25,85,86]. Studies have demonstrated that the emission of volatiles by *B. bassiana* can attract *Anopheles stephensi* mosquitoes [87]. Furthermore, deploying black cloths impregnated with *M. anisopliae* or *B. bassiana* in mosquito traps has shown significant reductions in the survival rates of female *Ae. aegypti*, and the inclusion of attractive lures to these traps can further enhance their effectiveness [88].

Hydrogel, a substrate for a granular formulation of fungal conidia, has been shown to attract gravid females under field conditions [16]. Methyl benzoate, derived from plants, acts as an insect semiochemical and exhibits larvicidal activity against mosquitoes [89]. The combination of *M. anisopliae* with Schinusole essential oil has demonstrated a synergistic effect against *Ae. aegypti* larvae [90]. Yeast volatiles are known to attract many insect species [91]. Inactivated yeast tablets lure have shown attractiveness to both *Ae. aegypti* and *Ae. albopictus* females and have been utilized in yeast-bait ovitraps [92]. Supplementation of sugar to *B. bassiana* conidia formulation can increase the attraction of *Ae. aegypti* and enhance their viability, resulting in a three-fold reduction in population [93]. Combining the oviposition attractant and larvicidal agents *B. thuringiensis israelensis* and *Bacillus sphaericus* in a single formulation can result in higher larval mosquito mortality [94]. Additionally, some bacterial or fungal secretions act as attractants and can affect mosquito behavior, such as oviposition strategy, egg hatching, development rate, and larval or pupa survival [95]. For example, Bt affects the oviposition strategy of *Ae. aegypti* and *Ae. albopictus* [96]. A sesquiterpene alcohol, cedrol, produced by *Fusarium falciforme* can affect the oviposition behavior of *An. gambiae* [97]. Therefore, combining oviposition attractants with fungal biopesticides can synergistically control mosquito adults as well as their aquatic larval offspring. The growing understanding and application of these mosquito attractants would contribute to optimizing lure-and-kill strategies and play a crucial role in integrated mosquito management programs.

### 4.4. Combined with Predators

The use of predators that feed on aquatic organisms has been demonstrated to be effective in controlling mosquito larvae [23]. Insects that have predatorial capacity to mosquito prey have been identified in the Orders Odonata, Coleoptera, Diptera (primarily aquatic predators), and Hemiptera (primarily surface predators) [98]. Among them, *Toxorhynchites* and copepods are the two most effective predatory organisms against mosquitoes [99]. It has been reported that many EPF have either no or very low impact on aquatic predators [100]. However, combined predator-parasite treatments have shown enhanced efficacy against mosquito compared to single-agent treatments. For example, the combination of *Metarhizium* with *Toxorhynchites* treatments drastically reduce lethal times of *Ae. aegypti* mosquitos compared to individual treatments [22,100,101]. The survival of adult *An. gambiae* exposed to *B. bassiana* after larval pre-exposure to a predator, namely nymphs of the dragonfly *Pantala favescens,* has been shown to increase the susceptibility of mosquito to fungal parasitism at the adult stage [102]. However, *A. flavus* displays a mortality rate of over 80% at dosage of 2 × 10^16^ (two-fold-higher dosage used in larval assays) when tested against two aquatic predators, *Alpheus bouvieri* and *Toxorhynchites splendens*, indicating that it cannot be directly applied directly to the aquatic region [70].

## 5. Engineering Manipulation of EPF to Improve Their Mosquitocidal Efficacy

With the advancement of genetic engineering techniques, genetic control methods have emerged as promising alternative strategies for enhancing the biological control capabilities of entomopathogenic fungi against mosquito vectors of disease [4]. Three strategies have been reported for modifying EPF.

### 5.1. Introducing Insecticidal Molecules into Mosquito

The insertion of insecticide expression genes into EPF can significantly enhance their mortality activity. For example, the genetic modification of *B. bassiana* expressing the Bt toxin *Cyt2Ba*, leads to a substantial improvement in its efficacy in killing mosquitoes [14]. Insecticidal activity can also be enhanced by expressing mosquito-inhibitory molecules. The *B. bassiana* strain expressing an *Ae. aegypti* trypsin-modulating oostatic factor (TMOF), which inhibits food digestion in the guts of adult and larval mosquitoes, exhibited increased virulence against *An. gambiae* compared to the wild-type strain [103]. By using specific fungal promoters to drive the expression of mosquito-killing genes in insect tissue, EPF can target and eliminate mosquitoes more accurately and efficiently [104]. The expression of ion channel blockers under the control of a hemolymph-specific promoter *Mcl1* in *Metarhizium* resulted in increased fungal lethality to mosquitoes at very low spore dosages, even as low as one conidium per mosquito [105]. In a semi-field assay conducted in Burkina Faso, an engineered *Metarhizium* strain expressing an insect-specific toxin (Hybrid) exhibited enhanced fungal lethality and a prolonged mortality effect compared to the wild-type strain, demonstrating its potential to synergistically manage insecticide-resistant mosquitoes in an endemic malaria area [17,106].

Another approach to increase mosquito-killing efficacy is by suppressing the host immunity. Expression of host miRNAs in *B. bassiana* has been shown to significantly enhance fungal virulence against insecticide-resistant mosquitoes. Engineered fungal entomopathogen *B. bassiana,* that produces host immunosuppressive miRNAs, can effectively suppress the host Toll immune response and facilitate fungal infection [107]. This pathogen-mediated RNAi (pmRNAi)-based approach provides an innovative strategy not only to enhance the efficacy of fungal insecticides but also to minimize the possibility of resistance development. Another alternative strategy for mosquito control is the combination of EPF and bacteria that express immune suppressive dsRNA. This combination has been shown to enhance the toxicity of EPF in leaf beetles by inhibiting host immunity [108]. In this strategy, microbiota in the mosquito gut can be modified and serve as a molecular adjuvant and immunomodulator against parasites when in combined application with EPF [109].

### 5.2. Introducing Antipathogen Effector to Block Vector Disease Transmission

To target the pathogen in mosquitoes is another strategy in genetic manipulation of EPF [110,111]. Genetically modified EPF strains can express antimalarial effector molecules and antimicrobial peptides. Recombinant *M*. *anisopliae* strains have been engineered to produce antimalarial effector molecules that inhibit the attachment of sporozoites to salivary glands, agglutinate sporozoites, or exhibit antimicrobial toxic activity to inhibit *Plasmodium* development. This approach resulted in a decrease of up to 98% in the malarial sporozoite count in mosquito salivary glands [112]. A similar strategy has also been achieved using midgut symbiont in mosquitoes. The paratransgenic control strategy, which involves expressing an antiplasmodial effector driven by blood meal induced (BMI) promoters, has proven to be effective in inhibiting pathogen infection [7,25,111,113].

### 5.3. Increasing the Fungal Tolerance to Adverse Environmental Conditions

For application in water, UV-B has no detrimental effect for sedimented conidia even no overlay of water [114]. However, when exposure of fungus-treated mosquito adults to sunlight, UV-B radiation can affect activity of conidia applied on the mosquito’s surface [115]. To enhance the efficacy of EPF, increasing their UV tolerance through genetic manipulation is another viable strategy. For instance, the expression of a photolyase from archaea in *M. robertsii* and *B. bassiana* has been shown to enhance their resistance to sunlight while maintaining their virulence against the malaria vector *An. gambiae* [116]. Genetic manipulation of other stress-tolerance-related genes, such as heat shock protein 25, can also improve thermal tolerance [117].

## 6. Conclusions and Future Prospect

Due to the ability to invade the insect from the cuticle and digestive tract, EPF have significant advantages in targeting mosquitoes at all life stages, including adults, eggs, pupae, and larvae [5,19,21,79]. Consequently, EPF have the potential to become the most promising and valuable biorational agents for mosquitoes in the future. Current mosquito control methods employ various mechanisms. The combination of EPF with different biocontrol strategies at different developmental stages can yield synergistic effects in mosquito control [7]. Six-month-long large-scale field study of a commercially In2Care Mosquito Traps, which combine a larvicide pyriproxyfen (PPF) with *B. bassiana*, has a better control efficacy than an IVM strategy consisting of source reduction, larviciding, and adulticiding for controlling *Ae. aegypti* eggs, larvae, and adults [24]. The integration of In2Care Traps or other EPF into the IVM program would have the great potential to become the most effective control strategy for mosquito control. Based on considering the compatibility, many strategies, such as chemical pesticide, attractants, bacterial insecticides, microbial metabolites and mosquito-symbionts can be integrated to enhance mosquito control effectiveness at different development stages [20,24,92].

However, EPF deployment and maintenance typically require more time and labor [24]. Therefore, it is crucial to enhance the effectiveness of EPF in mosquito control to reduce the frequency of application and the associated maintenance costs. Serial passage of EPF has demonstrated the ability to modify virulence and host specificity, Furthermore, when EPF is passaged through an insect host, it can enhance its virulence [63,118]. For the future development of EPF, it is crucial to focus on screening and artificial breeding of mosquito-killing fungi or species-specific strains that exhibit high efficiency in controlling mosquitoes at different life stages, elucidate their pathogenic mechanisms, and identify the factors that influence their efficacy at each infection step.

Genetic engineering of fungi to enhance mosquito-killing efficiency is a promising strategy. However, it also raises concerns about environmental or biosafety issues, such as the potential release releasing drug resistant gene and host specificity. Currently, commonly used selection markers for engineered fungal strains include herbicides and amphotericin B, but the release of these drug resistance genes into the environment is prohibited to prevent their dissemination [119,120]. Avoiding the use of drug-resistant gene-based selection markers has become a major challenge that hampers the widespread application of genetically engineered organisms. Therefore, the development of marker-free genetic transformation techniques and biorational selection markers, such as nutrition-metabolism-related genes becomes unavoidable [119,121,122]. Moreover, employing tissue-specific promoters, such as the hemolymph-specific promoter *Mcl1*, to regulate gene expression is an adoptable approach for reducing the public risk associated with genetically modified strains [104]. Present studies indicate that genetic engineering manipulation of EPF by expression of exogenous toxin in insect hemolymph does not alter host range, as host recognition occurs during the penetration step [123]. Construction of highly efficient genetically engineered fungal strains using these techniques will play a significant role in the future application and promotion of entomopathogenic fungi in integrated mosquito management.

## Figures and Tables

**Table 1:** Recent application and effectiveness for EPF in biocontrol of mosquito.

Fungal Strain	Stage	Mosquito Species	Mosquito Killing Effect (Mortality) *	Reference
*M*. *humberi*	Egg	*Ae. aegypti*	25–30% eclosion	[29]
*M*. *anisopliae*	Pupae	*Ae. aegypti*	1 × 10^7^ conidia/mL: 43%/24 h; 77%/48 h	[33]
	Larvae	*Ae. aegypti*	1 × 10^8^ spores/mL: ST_50_ = 48 h	[43]
	Larvae	*Cx. pipiens*	1 × 10^8^ conidia/mL: 88%; LT_50_ = 22.6 h	[55]
	Larvae	*Cx. qinquefasciatus*	1 × 10^6^–1 × 10^10^ conidia/mL; LT_50_ = 3.25 d	[47]
	Larvae	*Ae. albopictus*	Pupation was delayed by 2.75%	[15]
	Larvae	*An. Stephensi*	Pupation was delayed by 83.3%	[12]
	Adults	*Ae. aegypti*	LC_50_ = 2.4 conidia/mL: ST_50_ = 5 d	[56]
	Adults	*An. gambiae*	1 × 10^11^ conidia/m^2^: 100%/7 d	[57]
	Adults	*An. stephensi*	1 × 10^7^ conidia/mL: 57.5%/ST_50_ = 10 d	[12]
*B*. *bassiana*	Pupae	*An. gambiae*	No effects on pupae	[58]
	Pupae	*Ae. albopictus*	2.5 × 10^8^ conidia/mL: 14.0–40.5%	[58]
	Larvae	*Ae. aegypti*	1 × 10^8^ conidia/mL: ST_50_ = 2 d	[43]
	Larvae	*Ae. albopictus*	1 × 10^6^ conidia/mL: LT_50_ = 3.68 d	[59]
	Larvae	*An. gambiae*	1.25–2.5 × 10^8^ conidia/mL: 97.2–100%	[58]
	Larvae	*An. stephensi*	1 × 10^6^–1 × 10^10^ conidia/mL: LT_50_ = 6.18 d	[47]
	Larvae	*Cx. pipiens*	1 × 10^8^ conidia/m:73.33%; LT_50_ = 38.35 h	[55]
	Larvae	*Cx. qinquefasciatus*	1 × 10^7^ conidia/mL; 36.47%/92 h	[60]
	Adults	*Ae. albopictus*	5 × 10^8^ conidia/mL: S_50_ = 5 d	[14]
	Adults	*Ae. aegypti*	1 × 10^8^ conidia/mL: 95%/11 d; LT_50_ = 4.5 d	[61]
	Adults	*Cx. pipiens*	1 × 10^8^ conidia/mL: LT_50_ = 7.9 d	[62]
	Adults	*An. coluzzii*	1 × 10^8^ conidia/mL: ST_50_ = 5–7 d	[63]
	Adults	*An. gambiae*	1 × 10^6^ spores/mL: LT_50_ = 5 d	[64]
	Adults	*An. stephensi*	1 × 10^6^ conidia/mL: LT_50_ = 4 d	[65]
*V. elodeae*	Pupae	*An. gambiae*	LC_50_ = 2.64 sfu/mL	[66]
*A. inflata*	Pupae	*An. gambiae*	LC_50_ = 5.486 sfu/mL	[66]
*C. eriocamporesii*	Eggs	*Ae. aegypti*	5 × 10^6^ conidia/cm^2^: 89% eclosion	[67]
	Larvae	*Ae. aegypti*	1 × 10^7^ conidia/mL: LT_50_ = 0.9 d	[67]
	Adults	*Ae. aegypti*	1 × 10^7^ conidia/cm^2^: LT_50_ = 18.2 d	[67]
*T. cylindrosporum*	Eggs	*Ae. aegypti*	1 × 10^5^ conidia/cm^2^: 85% eclosion	[68]
*A. parasiticus*	Larvae	*Ae. Aegypti*	LC_50_ = 1.0 × 10^7^ conidia/mL; 24 h	[69]
			LC_50_ = 2.99 × 10^5^ conidia/mL; 48 h	
*A. flavus*	Larvae	*Ae. aegypti*	2 × 10^8^ conidia/mL: >90%	[70]
*T. asperellum*	Larvae	*Aedes* spp.	2.68 × 10^8^ conidia/mL; LT_50_ = 12.33 h	[71]
*C. clavisporus*	Larvae	*An. stephensi*	1 × 10^6^ conidia/mL: LT_50_ = 1.3 d	[9]
*A. clavatus*	Larvae	*Cx. quinquefasciatus*	0.5–2.5 × 10^8^ spores/mL: 17.0–74.3%/48 h	[9]
		*Ae. aegypti*		
		*An. gambiae*		
*C. macrosporus*	Larvae	*Ae. aegypti*	8.3 × 10^4^ conidia/cm^2^: 100%/72 h	[72]

* LT_50_: Median Lethal Time; ST_50_: Median Survival Time; LC_50_: Median Lethal Concentration.

## Data Availability

Not applicable.

## Supplementary Materials

The following supporting information can be downloaded at: https://www.mdpi.com/article/10.3390/jof9070746/s1, Table S1. Recent research of fungal metabolites 35 in mosquito control. References [46,48,49,70,71,79,82,83,84,124,125,126,127,128,129] are cited in the supplementary materials.
