# Supplementary material for: Recent Advancements in Pathogenic Mechanisms, Applications and Strategies for Entomopathogenic Fungi in Mosquito Biocontrol
Source: J Fungi (Basel). 2023 Jul 13;9(7):746. doi: 10.3390/jof9070746 (PMC10381795; doi:10.3390/jof9070746)
Supplement: Supplementary file 1 [file jof-09-00746-s001.zip › jof-2496420-supplementary.pdf]

Review

Recent Advancements in Pathogenic Mechanisms, Applications and Strategies for Entomopathogenic Fungi in Mosquito Biocontrol

Yujie Qin [1,2,3], Xiaoyu Liu [1,2,3], Guoxiong Peng [1,2,3], Yuxian Xia [1,2,3] and Yueqing Cao [1,2,3,*]

[1] School of Life Sciences, Chongqing University, Chongqing 401331, China
[2] Chongqing Engineering Research Center for Fungal Insecticides, Chongqing 401331, China
[3] Key Laboratory of Gene Function and Regulation Technologies under Chongqing Municipal Education Commission,401331, Chongqing, China
* Correspondence: yueqingcao@cqu.edu.cn

Table S1 Recent research of fungal metabolites in mosquito control

Fungi	Target	Species	Extraction agent	Effective ingredient	Reference
Aspergillus terreus	Larvae<br>Pupae	*Anopheles stephensi Culex quinquefasciatus Aedes aegypt*	Ethyl acetate<br>Methanol	n-hexadecanoic acid; methyl 12,15-octadecadienoate; butyl 9-hexadecenoate; bis(hex-5-en-1-yloxy) (dimethyl)silane; 4-(2,3-imethoxybenzylidene)-3-methyl-1-(4-nitrophe_x005f nyl)-2-pyrazolin-5-one; and 1,4 epoxynaphthalene-1(2H)-methanol, 4,5,7-tris(1,1-dimethylethyl)-3,4-dihydroo	[49]
Metarhizium anisopliae	Larvae<br>Pupae<br>Adult	*Anopheles stephensi Aedes aegypti*<br>*Culex quinquefasciatus*	Ethyl acetate	camphor; caprolactam; and monobutyl phthalate	[48]
Metarhizium anisopliae	Larvae	*Aedes aegypti*<br>*Culex quenquefacistus*	_	Cell free culture filtrates*	[124]
Penicillium toxicarium	Larvae<br>Adults	*Anopheles gambiae*	Ethyl acetate		[82]
Aspergillus lateralis; Cladosporium; Trichoderma Diaporthe; Albifimbria Emmia; Sarocladium	Larvae	*Aedes aegypti*	Ethyl acetate<br>isopropanol		[125]

Aspergillus niger	FREP1 protein of Plasmodium	*Anopheles gambiae*		*P*-orlandin	[83]
Fusarium oxysporum	Larvae<br>Pupae	*Anopheles stephensi Aedes aegypti*<br>*Culex quinquefaciatus*	Ethyl acetate	carbonic acid, bis (1-methylethyl) ester; 4-heptanol, 2-Methyl; and 1-4-benzopyram-4-one-5, 6, 7, 8-tetrahydro-3-hydroxy-2-methyl	[46]
Penicillium spp.	Larvae	*Aedes aegypti* L<br>*Culex quinquefasciatus*	Ethyl acetate		[126]
Trichoderma asperellum	Larvae	*Anopheles*	Methanolic extract	2,3-di hydro thiopene; p-cymene; alpha pinene; hexadecanoic acid; 8-methyl quinoline; (Z, Z)-9,12-octa decadienoic acid, methyl ester; and 2,3-dihydro-3,5-dihydroxy-6-methyl-4H-Pyran-4-one	[71]
Aspergillus tamarii	Larvae	*Aedes aegypti*<br>*Culex quinquefasciatus*	Ethyl acetate	preg-4-en-3-one, 17. α-hydroxy-17. β-cyano-; Trans-3-Undecene-1,5-diyne; and pentane, 1,1,1,5-tetrachloro-	[127]
Penicillium daleae	Larvae	*Culex quinquefasciatus Aedes aegypti*	Ethyl acetate		[128]
Beauveria bassiana	Larvae	*Culex quinquefasciatus*	Ethyl acetate	N-hexadecanoic acids; Z, Z-9,12 octadecadienic acid; 9-eicosyne; heptacosane; tetrateracontane; and 7 hexyleicosane	[46]

Penicillium spp.	Larvae	*Culex quinquefasciatus*	Ethyl acetate	Propanoic acid, ethyl ester; Acetic Acid, Propyl Ester; Isopentyl Acetate; Acetic Acid, 2-Methylpropyl Ester; Behenic alcohol; 1-Hexadecene; 1-Octadecene; 1-Hexacosanol; N-Hexadecanoic acid; 1-Tetradecanol; 1-Dodecene; Tetrydamine; and Octadecanoic acid	[129]
Trichoderma longibrachiatum; *Trichoderma viride*	Larvae	*Aedes aegypti* *Aedes albopictus*	_	Cell free culture filtrates*	[84]
Aspergillus spp.	Larvae	*Aedes aegypti*	Ethyl acetate	Aspergillol B	[79]
Aspergillus flavus	Larvae	*Aedes aegypti*	-	aflatoxins (B1, B2, G1 and G2)	[70]

* Cell free culture filtrate: no extraction agent
